# The pattern of paediatric otorhinolaryngological disorders seen at the Rivers State University Teaching Hospital, South-south Nigeria: a 3-year review

**DOI:** 10.11604/pamj.2022.42.94.31889

**Published:** 2022-06-03

**Authors:** Datonye Christopher Briggs, Victor Ohaka Ikenga, Ureh Annabel Oparaodu, Ebong Mbak

**Affiliations:** 1 Department of Paediatrics, Rivers State University Teaching Hospital, Port Harcourt, Nigeria,; 2 Department of Otolaryngology (ENT), Rivers State University Teaching Hospital, Port Harcourt, Nigeria

**Keywords:** Diseases, paediatrics, epidemiology, Rivers State, Nigeria

## Abstract

**Introduction:**

otolaryngological disorders vary among children due to diverse underlying aetiologies and pathologic processes.This study audits the pattern of paediatric ear, nose and throat diseases seen at the Rivers State University Teaching Hospital.

**Methods:**

a cross-sectional retrospective review of medical records of children (aged 0 - 17 years) seen between 1^st^ January 2018 and 31^st^ December 2020 were retrieved and analysed using IBM SPSS version 25.0. Results were presented as frequencies and percentages for categorical variables and mean and standard deviation for continuous variables.

**Results:**

a total of 5,533 paediatric visits were documented over the study period, making up 36.7% of all patients seen. There were 2,516 completed paediatric medical records. Males slightly predominated, accounting for 1,369 (54.5%), mean age was 6.77 years (SD ± 5.10) and ranged 2 weeks to 17 years. Age groups 0-4 years were the most affected. Ear disorders were the commonest disorders found (1637, 65.1%), followed by throat/neck disorders (650, 25.8%) and then nose disorders (229, 9.1%). The top 2 disorders based on regions were as follows: Cerumen Auris (426, 16.9%) and Otitis Externa (252, 10.2%) for ear disorders; Chronic / Allergic rhinosinusitis (107, 4.3%) and foreign body in the nose (72, 2.9%) for nose disorders and Adenotonsillar hypertrophy (544, 21.6%) and speech disorders (23, 0.9%) for throat disorders respectively.

**Conclusion:**

among the paediatric group of patients, ear disorders predominated. Cerumen Auris, Adenotonsillar hypertrophy and chronic/allergic rhinosinusitis were the commonest ENT disorders.

## Introduction

A diverse pattern of otorhinolaryngological disorders present to the ENT surgeon every day, the world over. These tend to vary even within the same locale and from one health institution to another based on their age group, gender, underlying aetiologies or pathologic processes and also on the availability of trained specialists and facilities for the diagnosis and treatment of such diseases [1,2]. Paediatric otorhinolaryngology is the study of disorders of the ear, nose and throat as they relate to the growth and development of head and neck structures [3] and remains an integral part of the specialty. Historically, even from the Victorian and Edwardian eras, Morell Mackenzie and Sir Felix Semon had a large paediatric ear, nose and throat practice which reported the existence of a wide variety of paediatric otorhinolaryngological disorders [4].

The paediatric age group constitutes almost half (46%) of the total Nigerian populace [5]. Albeit, only a few studies are available which report that ear, nose and throat related disorders among children and adolescents contribute substantially to the burden of otorhinolaryngological consults in ENT clinics across Nigeria, ranging from about 39.6% [6] to 41% [2]. Although paediatric ear, nose and throat disorders are not among the leading causes of paediatric mortality, the focus of various existing health policies for children and adolescent population undermines the substantial morbidity and impact on health-related quality of life that arise from the ear, nose and throat diseases [2,7]. Still, ear nose and throat related complaints are a major cause for a paediatric consult to the doctor in resource-limited countries [8-10].

Otorhinolaryngological problems in children could be congenital, infective, traumatic or neoplastic. Congenital disorders include pinna and external auditory canal malformations, pre-auricular sinus, choanal atresia. Infective conditions include adenotonsillitis, otitis (externa and media), rhinosinusitis, pharyngitis and laryngitis [2]. Therefore, an in-depth understanding of the pathophysiological mechanism of conditions and patterns in various settings will lead to better management of these patients. Studies on paediatric otorhinolaryngological disorders are scanty in our environment. The objective of this study, therefore, was to undertake an audit of the ear, nose and throat disorders seen among children and adolescents presenting to the ENT outpatient clinic in the Rivers State University Teaching hospital as this will serve as a template to improve the ENT-related services for this age category of the populace.

## Methods

**Study design:** this was a retrospective cross-sectional study.

**Study setting:** the study was conducted at the Ear Nose and Throat (ENT) department of the Rivers State University Teaching Hospital, Port Harcourt, Rivers State, South-South, Nigeria between 1^st^ January 2018 and 31^st^ December 2020. This institution is one of the few that provides tertiary level health care services and training in the South-Southern region of Nigeria. All paediatric cases presenting directly to the ENT clinic or who were referred either from the paediatric out-patient clinic or family medicine clinics and managed in the unit within the study period were included in the study. The ENT clinic is administrated by a team of Consultant ENT surgeons / ENT trained nurses and hosts about 30 to 44 patients per day and 120 to 240 new patients per month - including children and adults.

**Participants:** all the case records of the patients that were managed for any otolaryngological condition and aged < 18 years during the three years under review were manually retrieved from the medical records and manually reviewed.

**Inclusion/exclusion criteria:** all patient records of children and adolescents under 18 years were included. Incomplete patient records or those with missing variables of interest were excluded.

**Data sources:** all patient case records are manually stored at the medical records of the institution and relevant patients´ characteristics are regularly entered manually by the attending ENT-trained nurses. There is no electronic database for medical records in our department, yet.

**Variables:** relevant data were extracted from these case records, using a purpose-designed proforma. These data included age, sex, diagnosis at presentation, treatment received, type of procedure(s) done during the period under review. The diseases were then grouped based on the three main regions of the complaints into the ear (otological), nose (rhinological) and throat (pharyngeal, laryngeal, oral cavity, head and neck) diseases.

**Statistical methods:** the data were analysed using IBM SPSS version 25.0 and results were displayed using descriptive statistics including frequencies and percentages. Chi-square test was used for categorical variables. All incomplete folder entries/ records with missing data or patients initially entered but duplicated following follow-up visits were excluded from the analyses.

**Ethical clearance:** permission was granted by the hospital management and head of department of ENT to carry out this review of medical records.

## Results

In this study period, a total of fifteen thousand and seventy-eight (15,078) patients were seen, managed and or followed up for an ear, nose or throat related disease at the department of ENT, RSUTH, Port Harcourt, Nigeria. Of these, five thousand five hundred and thirty-three (5,533) were in the paediatric age range and accounted for (36.7%) of all the patient- attendees seen at the ENT clinic in the 3-years under review. On average about 33 patients accessed the ENT clinic daily and 149 new patients (adults and children) were seen on a monthly with otorhinolaryngological-related diseases. After excluding all follow-up visits, a total of 2,812 paediatric case records were assessed, of which an additional 296 were excluded for incomplete data. Hence, a total of 2,516 paediatric ENT cases were eligible for analysis. The mean age of the patients was 6.77 years (SD ± 5.10) and ranged from 2 weeks to 18 years. There was a slight male preponderance of 1,369 (54.4%) (p=0.16) with a M: F = 1.19: 1. However, there is no significant difference in the male to female ratio of clinic attendance as seen in Table 1.

**Table 1:** gender distribution of the children attending ENT clinic by year

Gender/Year	2018		2019		2020		Total	
	N	%	N	%	N	%	N	%
**Female**	207	8.2	541	21.5	399	15..9	1147	45.6
**Male**	216	8.6	692	27.5	461	18.3	1369	54.4
**Total**	423	16.8	1233	49.0	860	34.2	2516	100.0

X2 =3.59; p-value= 0.16

Across the three years under review, the age group 0-4 years were the most affected with a total number of 1100 patients, constituting 43.7% of the entire paediatric age group whereas the 15 - 18 years age group were the least affected and accounted for 275 (10.9%) of the paediatric age group, as seen in Table 2. When all ENT disease categories were compared by year, the Ear (Otologic) disorders were the most commonly occurring disease and was seen in 1,637 of the patients accounting for 65.1% of all paediatric cases presenting with ENT disorders. This was followed by throat disorders 650, 25.8% and then, nose disorders (229, 9.1%). This was statistically significant (p=0.005) when compared to the other ENT disorders, as depicted in Table 3.

**Table 2:** age distribution of the children attending ENT clinic by year

Age/Year	2018		2019		2020		Total	
	N	%	N	%	N	%	N	%
**0-4**	195	7.8	561	22.3	344	13.7	1100	43.7
**5-9**	116	4.6	326	13.0	219	8.7	661	26.3
**10-14**	80	3.2	228	9.1	172	6.8	480	19.1
**15-18**	32	1.3	118	4.7	125	5.0	275	10.9
**Total**	423	16.8	1233	49.0	860	34.2	2516	100.0

X2 =21.9; p-value= 0.001

**Table 3:** distribution of ENT disease categories of the children by year

Disorder category/year	2018		2019		2020		Total	
	N	%	N	%	N	%	N	%
**Ear**	254	10.1	802	31.9	581	23.1	1637	65.1
**Nose**	31	1.2	125	5.0	73	2.9	229	9.1
**Throat**	138	5.5	306	12.2	206	8.2	650	25.8
**Total**	423	16.8	1233	49.0	860	34.2	2516	100.0

X2 =14.98; p-value= 0.005

**Figure 1:**
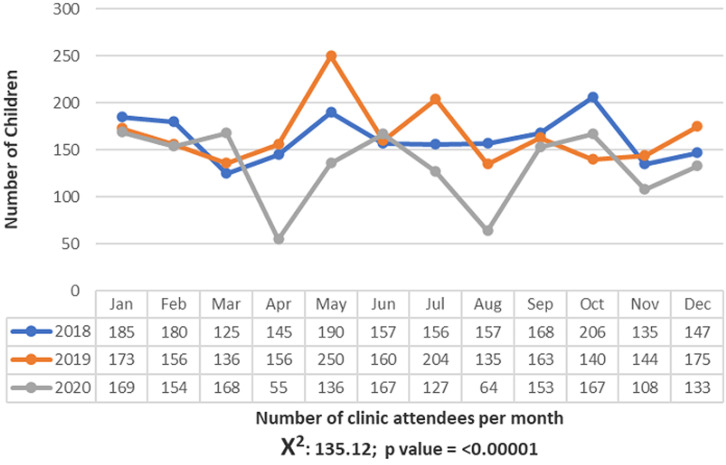
trend in paediatric ENT clinic attendance between 2018 and 2020

The 0-4-years group of paediatric patients were the most affected across all categories of ENT disorders with 585 (23.3%) presenting with Ear disorders, 81 (3.2%) Nose disorders and 434 (17.2%) throat disorders. The 15 - 18 years age group were the least affected and the difference across the age groups was statistically significant (p=< 0.0001) as seen in Table 4. Overall, males had significantly more ear, nose and throat disorders when compared to females, the margin being widest among disorders of the throat, as seen in Table 5. With regard to the pattern of paediatric ENT disorders as shown in Table 6, Cerumen auris as seen in 426 (16.9%) of the children were found to be the most common ear disorder, followed by Otitis Externa (252, 10.2%); Otomycosis (213, 8.5%); Acute Otitis Media (195, 7.9%) and Chronic Suppurative Otitis Media (148, 5.9%). Chronic / Allergic rhinosinusitis (107, 4.3%), foreign body in the nose (72, 2.9%) and acute rhinitis (15, 0.6%) were the top -3 most common disorders of the Nose. Whereas, Adenotonsillar hypertrophy (544, 21.6%), speech disorders (23, 0.9%), Acute tonsillitis (17, 0.7%), pharyngitis (14, 0.6%) and ankyloglossia (10, 0.4%) were among the top 5 disorders of the throat seen in the children in this study. The pattern of paediatric otolaryngological clinic attendance between 2018 and 2020is displayed in Figure 1.

**Table 4:** distribution of children according to age and ENT disorder

Age (years)	Ear disorders		Nose disorders		Throat disorders		Total	
	N	%	N	%	N	%	N	%
**0-4**	585	23.3	81	3.2	434	17.2	1100	43.7
**5-9**	492	19.6	46	1.8	123	4.9	661	26.3
**10-14**	346	13.8	60	2.4	74	2.9	480	19.1
**15-18**	214	8.5	42	1.7	19	0.8	275	10.9
**TOTAL**	1637	65.1	229	9.1	650	25.8	2516	100

X2 =218.5; p-value= <0.0001

**Table 5:** distribution of children according to sex and ENT disorder

SEX	EAR	NOSE	THROAT	TOTAL
	N	N	N	N
**Female**	779 (47.6%)	109 (47.6%)	259 (39.8%)	1147(45.6%)
**Male**	858(52.4%)	120(52.4%)	391 (60.2%)	1369(54.4%)
Total	1637(100%)	229(100%)	650(100%)	2516(100%)

X2 =11.649; p-value=0.003

**Table 6:** pattern of paediatric ENT disorders

Disorder	Diseases	Frequency	Percentage
**EAR**	Chronic Suppurative Otitis Media	148	5.9
	Acute Otitis Media	195	7.9
	Otitis Externa	257	10.2
	Otitis Media with Effusion	8	0.3
	Aural polyps	64	2.5
	Tympanic membrane perforation/ EUC Injuries	35	1.4
	Eustachian tube disorders	20	0.8
	Hearing loss	83	3.3
	Tinnitus	14	0.6
	Cerumen Auris	426	16.9
	Foreign body in the ear	93	3.7
	Otalgia	19	0.8
	Bell´s palsy	4	0.2
	Periauricular sinus/abscess	20	0.8
	Ear Atopy	7	0.3
	Acute Mastoiditis	2	0.1
	Aural Keloids	5	0.2
	Otomycosis	213	8.5
	Aural trauma	11	0.4
	Other ear disorders	13	0.6
**NOSE**	Acute rhinitis	15	0.6
	Chronic /allergic rhinosinusitis	107	4.3
	Epistaxis	9	0.4
	Foreign body in the nose	72	2.9
	Nasal polyps	8	0.3
	Sino-nasal tumours	5	0.2
	Maxillary sinusitis	3	0.1
	Nasal / nasal septal injuries	4	0.2
	Angiofibroma of nasopharynx	2	0.1
	Other nasal disorders	4	0.2
**THROAT**	Adenotonsillar hypertrophy	544	21.6
	Acute tonsillitis	17	0.7
	Pharyngitis	14	0.6
	Ankyloglossia	10	0.4
	Speech disorders	23	0.9
	Papilloma of the tongue	2	0.1
	Cleft palate	1	0.0
	Maxillary sinus cyst	2	0.1
	Sublingual cyst	2	0.1
	Foreign body ingestion	4	0.2
	Cervical lymphadenopathy	6	0.2
	Laryngomalacia	5	0.2
	Thyroglossal cyst	3	0.1
	Submandibular sialoadenitis	1	0.0
	Cystic hygroma	3	0.1
	Mumps parotitis	4	0.2
	Others	10	0.4
	**TOTAL**	**2516**	**100**

## Discussion

This study demonstrated that the paediatric group (0-17 years) constituted 36.7% of the total population of otolaryngological patient clinic visits at the Rivers State University Teaching Hospital and similar to the report by Onotai et al [6] in another tertiary hospital in Port Harcourt, Rivers State, were 39.6% of the patients seen in their 5-year review were children aged 0-17 years. Overall, out of the 2516 paediatric case records reviewed, males slightly predominated accounting for 1,369 (54.5%) and with a male: female ratio of 1.19: 1. This also meant more boys than girls attended the ENT clinics. However, there was no statistically significant difference in the male to female ratio of clinic attendance in the 3-years under review. Our finding was comparable to reports by other authors in Port Harcourt [6] and Ibadan [2], in Nigeria, Zambia [11] and Nepal [12], India [13] and Bangladesh [14] in Asia, respectively. The age group 0-4 years were the most affected of the entire paediatric population irrespective of the year, accounting for 1100 (43.7%). This was similar to the findings by Ibekwe and Mbalaso in an earlier study done in Port Harcourt, Rivers State. However, our finding was not consistent with those from other studies where the authors reported age categories being affected differently depending on the specific ENT related disorder. For instance, in the study by Sigdel and Nepali [15], the 6 -10 years-age group was the most affected group presenting with ear disorders whereas in another study by Kamfwa and Mwanakasale 5 to 14 year- age categories in Zambia [11]. Our finding may be as a result of the fact that parents tend to closely monitor children below five years of age and would rather seek care when these “essential parts” of the body are affected or are diseased as was similarly found in a study by Villarreal and colleagues [16]. Also, there is the possibility of an increased occurrence of ear-related infections among under-fives in Port Harcourt who are both in day-care and nursery schools as reported in another study [17], necessitating presentation at the hospital.

Overall, in this study, we observed that most of the children presenting to the ENT department had mostly ear-related disorders accounting for more than two-thirds (65.1%) of all the cases. This was comparable with findings from an earlier study in another tertiary facility in Port Harcourt, which reported that ear-related disorders accounted for 65.33% of the paediatric cases. Although lower than ours, other authors in Nigeria similarly report ear-related disorders as the most common ENT disorder and include 8.2% in Enugu [18], 22.4% in Kano [19], 28.5% in Ibadan [2] respectively. Likewise, authors from other countries reported that ear disorders were predominant among their paediatric cohorts such as 39.75% in Dhaka [14], 47.1% in Zambia [11], 49.32% in India [13] and 57.84% in Nepal [15]. Our finding however compared unfavourably with the study by Tall *et al*. in Senegal, where nose disorders were reported to be the most common paediatric ENT disorder, accounting for 54.6% of the cases. Their finding, albeit, was because authors included adenoidal hypertrophy as a disorder of the nose. Furthermore, in this study, it was observed that the 0-4 year-age group were most affected by ear-related disorders, which was similarly reported in a study in Nepal [12] but contrast findings from the study by Kamfwa and Mwanakasale [11] in Zambia where the 5 - 14 years-age group accounted for most of the ear-related disorders.

In this study, we report that the commonest ear-related disorders were cerumen auris (16.9%), followed by otitis externa (10.2%) and otomycosis (8.7%). Our finding of cerumen auris as the commonest ear-related disorder was, although lower, similar to a study in Port Harcourt [20] that found cerumen auris accounting for 18.34%. Also in Ibadan [21], cerumen auris contributed the most (25.8%) among primary school children aged 5 - 13 years and among a cohort of deaf students aged between 5 and 38 years cerumen auris was the most common ear-related disorder and accounted for 15.5% of the entire group, of which 79% were in children aged 5 - 19 years. Other authors outside Nigeria also found cerumen auris to be the predominant ear-related disorder with a prevalence of 33.4% to 40.9% in Nepal [15,22], 28.8% in Zambia [11] and 23.4% in Tanzania [23]. The finding in this study, however, contrasts reports from other Nigerian studies that found chronic suppurative otitis media as the commonest ear-related disorder [6,18]. The plausible explanation for our finding is most likely the routine practice by mothers to clean the ears of their infants and children with earbuds which inadvertently alters the ears´ physiologic mechanisms that eventually lead to wax impaction, as has been similarly reported in other studies [20-24]. Similarly, several other authors [23,25] have described that the practice of frequent cleaning of the ears with buds pushes in the cerumen deeper into the external auditory canal which then cannot be removed by the physiologic ‘outward migration’ of the epithelium of the canal.

Otitis externa (10.2%) and otomycosis (8.5%) ranked the second and third-commonest ear-related disorder noted in this study. While reasons are not obvious as to the causes of otitis externa and otomycosis, our rather high prevalence among mostly the under-fives may probably be linked to the habitual practice of cleaning children´s ears with cotton buds as a way to remove cerumen auris and has been similarly reported in a study by Nussinovitch and colleagues [26]. The skin of the external auditory meatus following unguided attempts to remove cerumen after using cotton buds can subsequently cause infection via trauma or maceration of the epithelial lining and pressure exerted by the cotton bud can also cause cerumen impaction [26]. Likewise, Otomycosis could occur following the trapping of moisture in the external auditory canal. Both ear disorders can then thrive when other factors such as high environmental temperatures and humidity are present [27]. This is the situation in our setting that may also contribute to the high prevalence of otitis externa and otomycosis in this study. Although otitis externa and otomycosis were not necessarily ranked among the top three ontological conditions in other settings, several authors found the prevalence of both otitis externa and otomycosis to be; 4.53% and 9.27% in Port Harcourt, Nigeria [20] and 8.6% and 4.7% in Nepal [22]; 5.8% and 0.9% in Zambia [11] respectively. In addition, other studies in Nigeria reported Chronic Suppurative Otitis Media (CSOM) and Acute Otitis Media (AOM) to be the leading causes of ear disorders; and ranged from 7.2% to 30.2% for CSOM [18,19] and 29.8% to 45% for AOM [2,18]. Nevertheless, we report a lower prevalence of 5.9% and 7.9% for CSOM and AOM respectively. Our findings may be due to the fact that most patients with symptoms like ear discharge and otalgia with or without a feverish condition are first reviewed by the paediatricians in the paediatric outpatient clinic and first treated with antibiotics for clinical suspicion of otitis media. Hence, the lower proportion of children presenting with more severe forms of ear infections like CSOM and AOM with or without effusion being referred to the ENT clinic.

Throat-related disorders were the second most common ENT disorders noted among the paediatric age group in this study, accounting for about a quarter (25.8%) of the cases. Obstructive adenotonsillar hypertrophy was the most common disorder (21.6%), followed by speech disorders (0.9%) and acute tonsillitis (0.7%). Our findings of adenotonsillar hypertrophy as the most common was consistent with what was previously documented by other studies in another centre in Port Harcourt, Nigeria [6,20]. On the contrary, authors in Ibadan found adenotonsillar hypertrophy to be the third most common ENT-related disorder. Also, our findings were not consistent with a study conducted in Senegal, where throat-related disorders were the third most common ENT disorder and acute tonsillitis (78.3%) was observed to be most predominant followed by goitre (6%) and pharyngitis (5.3%). Several other authors within Africa and Asia found adenotonsillar hypertrophy to range from 6.51% to 18.1% [11-14]. Throat disorders as a whole were also more common among the 0-4 years-age group and were similar to what was observed in Sudan [10] Senegal [28], Nepal [12], India [13]. Our findings differed from other studies, which reported the 10-16 years-age group as being the more affected by throat disorders [12,13].

Chronic/allergic rhinosinusitis (107, 4.3%) and foreign body in the nose (72, 2.9%), were the top-ranked nose-related disorders observed in this study. This was similar to what was reported by Kwamfa and Mwanakasale, in Zambia which found foreign bodies in the nose and rhinitis as the top-ranked nose-related disorders, however, their prevalence was higher, contributing 27% and 15.1%. Also, findings from other authors in Nigeria reported rhinosinusitis to account for nose-related disorders ranging from 1.7% (in Ibadan) to 18.4% (in Port Harcourt) [2,6]. The common predisposing factors for chronic rhinosinusitis among children include viral upper respiratory tract infection and acute exacerbation of allergic rhinitis. Also being, in a highly industrialised setting like Port Harcourt with a high rate of air pollution that could affect the respiratory airways may also be another reason for our findings, as has been similarly reported in another study in Port Harcourt [6]. Unsurprisingly, children being very inquisitive and being within the age range of exploring their bodies and environment are at increased risk of instilling foreign objects into their nostrils and other indeed other orifices like the ears and mouth. In this study, we report foreign body in the nose to be the 2nd most common reason for paediatric ENT clinic visit following a nose-related disorder. Foreign bodies ranging from beads to pebbles were the most common objects found and removed. Our prevalence of 2.9% were lower than the 27% reported in a study in Zambia but higher than the 1.4% reported by another study in Ibadan. In this 3-year review, there was a significant decline in paediatric ENT clinic attendance. Marked reductions were observed in April and August 2020 and these coincided with the periods of COVID-19 lockdown with near-total restriction of movement within the state. Also, trends reveal a slight decrease in the total number of clinic attendees in the year 2020, even after bans had been lifted. This may be a reflection of increasing service charges and overall aversion to seeking care in the facility as many patients believed they would contract COVID-19 by visiting the hospital. Hence, many may have resorted to self-help or alternative care.

*Limitation:* our study is limited by its retrospective design and the fact that poor recording keeping resulted in some records being excluded and also, study findings only reflect the paediatric population attending the ENT clinic of our hospital. Albeit, the centre services children from most of the southern states.

## Conclusion

Among the paediatric group of patients, ear disorders predominated. Cerumen Auris, Adenotonsillar hypertrophy and chronic/allergic rhinosinusitis were the commonest ENT disorders.

### What is known about this topic

- A diverse pattern of otorhinolaryngological disorders present to the ENT surgeon every day and these vary from one health institution to another, within the same locale, differ by age group, gender, underlying aetiologies / pathologic processes and also vary based on the availability of trained specialists and facilities for the diagnosis and treatment of such diseases;
- Ear nose and throat related complaints are a major cause for a paediatric consult to the doctor in resource-limited countries.

### What this study adds

- Ear-related disorders are the commonest in the Rivers State University Teaching Hospital followed by throat and then nose-related disorders;
- The under-five paediatric age group are the most affected across all ENT related disorders in our setting;
- COVID-19 restrictions in the State resulted in significantly reduced ENT clinic visits.
